# Supplementary material for: Mapping of Low-Frequency Raman Modes in CVD-Grown Transition Metal Dichalcogenides: Layer Number, Stacking Orientation and Resonant Effects
Source: Sci Rep. 2016 Jan 14;6:19476. doi: 10.1038/srep19476 (PMC4725868; doi:10.1038/srep19476)
Supplement: Supplementary Information [file srep19476-s1.pdf]

Supplementary Information - Mapping of Shear and Layer-Breathing Raman modes in CVD-Grown Transition Metal Dichalcogenides: Layer Number, Stacking Orientation and Resonant Effects

Maria O'Brien[1,2†], Niall McEvoy[1,2†*], Damien Hanlon[2,3], Toby Hallam[2,3], Jonathan N. Coleman[2,3] and Georg S. Duesberg[1,2*]

[1]School of Chemistry, Trinity College Dublin, Dublin 2, Ireland

[2]Centre for Research on Adaptive Nanostructures and Nanodevices (CRANN) and Advanced Materials and BioEngineering Research (AMBER) Centre, Trinity College Dublin, Dublin 2, Ireland

[3]School of Physics, Trinity College Dublin, Dublin 2, Ireland

†These authors contributed equally.

Additional MoS_2 Analysis

Peak position maps for A'_1/A_{1g} and $E'/E_g/E^1_{2g}$ modes are shown in Figure S1(a) and (b), respectively. A plot of the Raman shift position as a function of layer number in Figure S1(c) shows clearly the red and blue shift in E'/E_g and A'_1/A_{1g} peaks, respectively as layer number increases, allowing an initial assessment of layer number to be made, as indicated in the optical image in Figure 2(a) in the main text. The corresponding shift in PL position as layer number increases, reflecting the changing band structure of MoS_2 with layer number, is illustrated in Fig S1(d) and (e), which show the position of the A1 and B1 exciton, respectively. The extracted PL spectra in Figure S1(f) show the A1 and B1 excitonic peaks at ~680 and ~640 nm respectively, consistent with previous reports for CVD-MoS_2[1, 2]. The enhanced intensity in monolayers is due to their higher luminescence quantum efficiency than few-layer flakes. Inset in Figure S1(f) shows the PL normalized to the maximum signal for each layer number, to further emphasize the changing PL (reflecting the changing electronic properties) with layer number. This highlights

the evolution in peak position and change in A:B exciton intensity ratio with changing layer thickness.

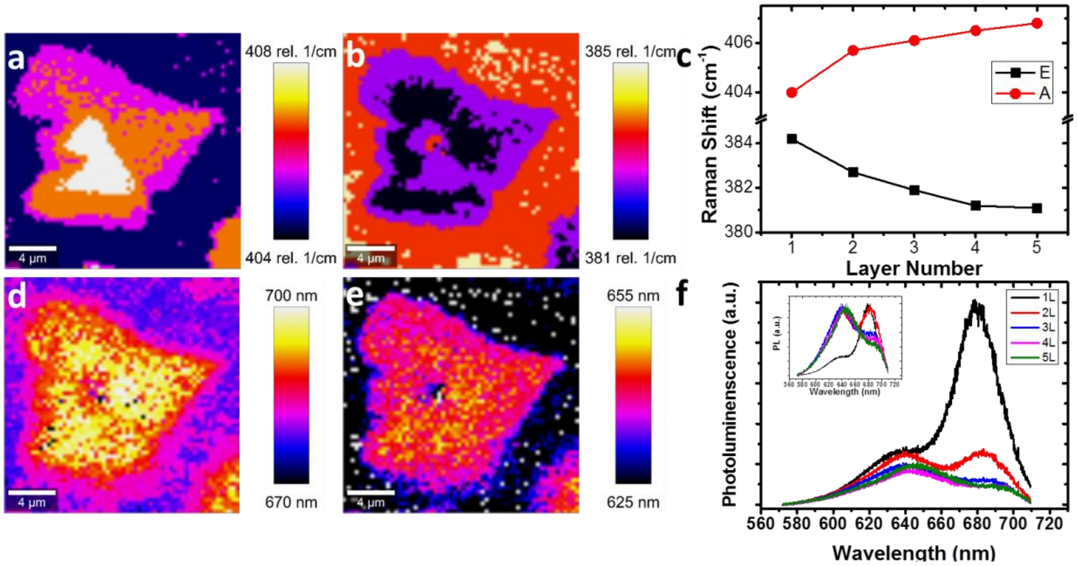

Figure S1 - Additional Raman and PL mapping of MoS_2 crystals of different layer thickness extracted from the regions shown in Figure 2(a) of the main text - (a) Map A'_1/A_{1g} mode position (b) Map of $E'/E_g/E^1_{2g}$ mode position (c) Plot of position of Raman shift for A'_1/A_{1g} and $E'/E_g/E^1_{2g}$ modes as a function of layer number. (d) Map of A1 exciton position (e) Map of B1 exciton position (f) PL spectra extracted from 1-5L regions corresponding to same areas as Raman spectra for each layer. Inset: Normalized version of the same spectra.

Additional $MoSe_2$ Analysis

Peak position maps for A'_1/A_{1g} and $E'/E_g/E^1_{2g}$ modes are shown in Figure S2(a) and (b), respectively. In the peak position maps for A'_1/A_{1g} in Figure S2(a), negligible variation is seen over the areas mapped, with the exception of expected decreases in the positions of grain boundaries[2], and variation at crystal edges, possibly due to doping or strain in these regions as has been reported for CVD-MoS_2[3, 4]. Figure S2(b) shows the peak position map for $E'/E_g/E^1_{2g}$, where a shift towards higher wavenumbers with decreasing layer numbers is observed. A slight blue-shift of $E'/E_g/E^1_{2g}$ is also seen at the edges of layers, likely due to edge and termination effects. Figure S2(c) shows a plot of Raman shift position for the E'/E_g and A'_1/A_{1g} modes as a

function of layer number, with a significant red shift observed for E'/E_g with increasing layer number, similar to MoS_2, but a less significant blue shift in A'_1/A_{1g} with increasing layer number. Figure S2(d) shows the map of B^1_{2g} mode position. This illustrates that this mode does not appear for monolayer, and while it is present for 2+L it does not vary significantly in position. The map of PL position for $MoSe_2$ is shown in Figure S2(e), showing a shift in position at the edge of each crystal, similar to previous observations for TMDs[3, 5]. The general shift in PL position for different regions is further shown in Figure S2(f), which shows the PL spectra extracted for the layer number regions discussed previously. The PL peak for monolayer occurs as single prominent maximum at a wavelength of ~800 nm, in line with previous reports of CVD and mechanically exfoliated $MoSe_2$[6, 7]. The shift in PL towards higher wavelengths (lower bandgaps) with increasing layer number is consistent with previous reports that show $MoSe_2$ and other 2D TMDs shifting to smaller and more indirect bandgaps with increasing layer numbers. Inset in Figure S2(f) shows the same PL spectra normalized to maximum PL intensity, in order to emphasize the shift in PL position with layer number.

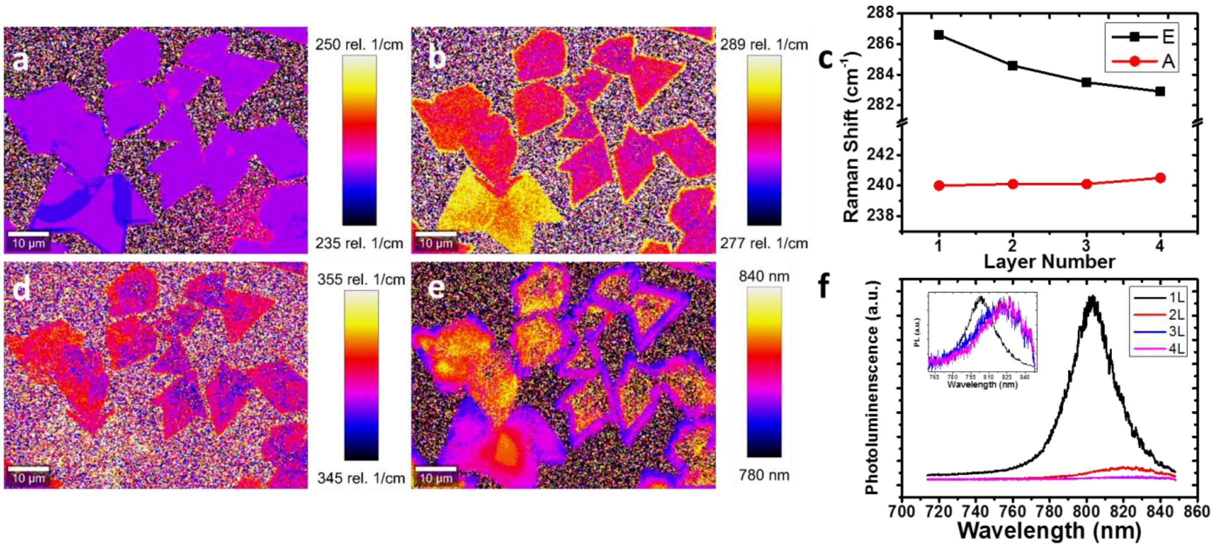

Figure S2 - Additional Raman and PL mapping of $MoSe_2$ crystals of different layer thickness extracted from the regions shown in Figure 4(a) of the main text - (a) Position map of maximum A'_1/A_{1g} mode intensity (b) Position map of maximum $E'/E_g/E^1_{2g}$ mode intensity (c) Plot of position of Raman shift for A'_1/A_{1g} and $E'/E_g/E^1_{2g}$ modes. (d) Position map of B^1_{2g} mode intensity (e) PL position map (f) PL spectra of $MoSe_2$ crystals of different layer thickness extracted from the regions shown in Figure 4(a) of the main text. Inset: Normalized version of the same spectra highlighting the evolution in peak position with changing layer thickness.

Evidence for changing stacking configurations is seen in the 4L low-frequency Raman spectra in Figure S3, where it is possible to identify a variety of 4L stacking configurations, which we have labelled A-H. The characteristic modes at ~11 cm^{-1}, 18 cm^{-1} and 24 cm^{-1} are mapped in Figure S3(a), (b) and (c), with labels for each spectrum extracted on the intensity scale for each. To facilitate identification of the layers in this area, an optical image of Figure 3(a) with enhanced contrast applied is shown here in Figure S3(d). By analysis of the Raman maps in Figure S3(a)-(c), it was possible to extract spectra A-H shown in Figure S3(e).

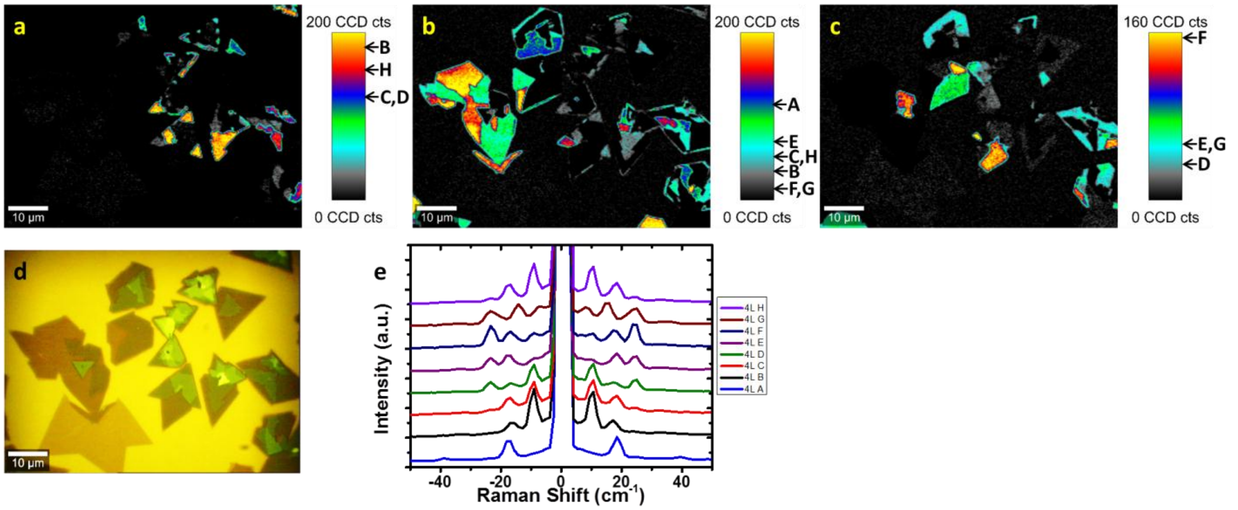

Figure S3 - Additional Raman mapping of $MoSe_2$ crystals of different layer thickness extracted from the regions shown in Figure 4(a) of the main text - (a) Peak intensity map for 4L $MoSe_2$ SM at ~11 cm^{-1} (b) Peak intensity map for 4L $MoSe_2$ SM at ~18 cm^{-1} (c) Peak intensity map for 4L $MoSe_2$ SM at ~24 cm^{-1} (d) Optical image of the region of $MoSe_2$ crystals mapped with additional contrast applied (e) Spectra of 4L $MoSe_2$ crystals of different stacking orientations extracted

In Figure S4, high-frequency Raman spectra for different stacking configurations are shown for the same regions for which each low-frequency spectrum was extracted. These spectra have been normalized to the A'_1/A_{1g} peak and offset for clarity. In Figure S4(a), the 2L-2H, -3R and -3R* spectra are shown, with no significant change in the spectra discernible between different stacking configurations. In Figure S4(b), the 3L spectra for differing stacking configurations are shown, with no major change in the spectra between different stacking configurations, however a small relative intensity change in B^1_{2g} mode can be observed in some of the 3L-3R-3R configurations. In Figure S7(c), the 4L spectra for differing stacking configurations are shown, with no observable change in the spectra between stacking configurations.

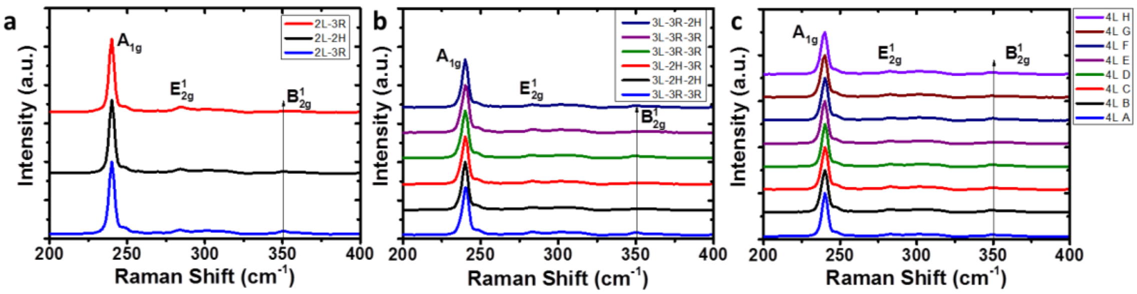

Figure S4 – Comparison of high-frequency Raman spectra for different stacking configurations within each layer number - (a) 2L high-frequency Raman spectra (b) 3L high-frequency Raman spectra (c) 4L high-frequency Raman spectra

Independent Assessment of Layer number with AFM

Much of the analysis in this work relies on the identification of layer number or stacking polytype for different TMDs using Raman spectroscopy. Atomic force microscopy (AFM) was used as an independent means of measuring the layer thickness, in order to verify the layer-number assignation from Raman spectroscopy and also to show that there was no change in thickness for different stacking polytypes of the same layer thickness. Figure S5 and S6 depict AFM analysis of two of the $MoSe_2$ regions analyzed in the main text. AFM measurements were carried out using a Veeco Dimension 3100 in tapping mode, with 40 N/m probes from Budget Sensors. In Figure S5(a) and (b), the maps of low-frequency maximum and position of maximum intensity have been reproduced from Figure 5 in the main text, along with the relevant optical image in Figure S5(c). Figure S5(d) shows an AFM image of this region with layer-thickness assignments from Raman spectroscopy overlaid; this has been rotated slightly in order to facilitate a direct comparison between AFM and Raman images. Figures S5(e-h) reproduce the low-frequency maps shown in Figure 5(e), (f), (h) and (i), in the main text, respectively. The centre triangle in Figure S5(g) is present due to overlap of peaks at this frequency for 3L with those of higher layer numbers. Figure S5(i) shows an AFM height map over this crystal region,

with several height profiles marked. These correspond to height profiles 1-5 in Figure S5. Height profile 1 shows a decrease in layer number from 3L to 2L. Height profile 2 shows no change in layer number across a 3L region, which contains several different stacking polytypes according to low-frequency Raman analysis. Height profile 3 shows an increase in layer number from a 2L region to a 3L region. Height profile 4 shows an increase in layer number from 0L (background) to a 2L region. Height profile 5 shows no increase in height across 2L regions of different stacking polytype, as shown through low-frequency Raman analysis, and then an increase on going from a 2L region to a 3L region. The height differences between different layers in each instance are labelled on each profile, and range from 0.9-1 nm per layer, comparable to previous reports of CVD grown TMDs[8, 9]. Decorative contamination can be seen in the AFM scans, which has been observed previously for CVD grown TMDs[2, 10] – this is likely due to preferential particle adsorption at the growth front, or atmospheric contamination post growth. These height profiles support the layer numbers previously identified by Raman spectroscopic measurements and further show the lack of any measurable change in height across different stacking polytypes within a given layer number. This illustrates that low-frequency Raman spectroscopy can identify features which cannot be observed using AFM alone.

Figure S6 shows further analysis of an area of CVD $MoSe_2$ analysed in the main text. In Figure S6(a) and (b), the maps of low-frequency maximum and position of maximum intensity of the relevant crystal have been reproduced from Figure 5 in the main text, along with the relevant optical image in Figure S6(c). Figure S6(d) shows an AFM image of this region with layer-thickness assignations from Raman spectroscopy overlaid, which has been rotated slightly in order to facilitate a direct comparison of AFM and Raman images. Figures S6(e-h) reproduce the low-frequency maps shown in Figure 6(e), (f), (h) and (i) in the main text, respectively.

Figure S6(i) shows an AFM height map over this crystal region, with several height profiles marked. These correspond to height profiles 1-5 in Figure S6. Height profile 1 shows an increase from 0L to 3L. Height profile 2 shows an increase from 2L to 3L. Height profile 3 shows no change across regions spanning different 3L stacking polytypes. It should be noted here that some interesting corrugations are present on the surface crossing height profile 3 which merit further investigation. Height profile 4 shows an initial increase from 2L to 3L, followed by no change in layer number across regions consisting of different 3L stacking polytype. Height profile 5 shows an increase from 0L to 3L and back down to 0L. Height profile 6 shows an initial increase of 2L, followed by an increase from 2L to 3L. The height differences between different layers in each instance are labelled on each profile, and range from 0.9-1 nm per layer, comparable to previous reports of CVD grown TMDs[8, 9]. These height profiles support the layer numbers previously identified by Raman spectroscopic measurements, and further show the lack of any measurable change in height across different stacking polytypes within a given layer number.

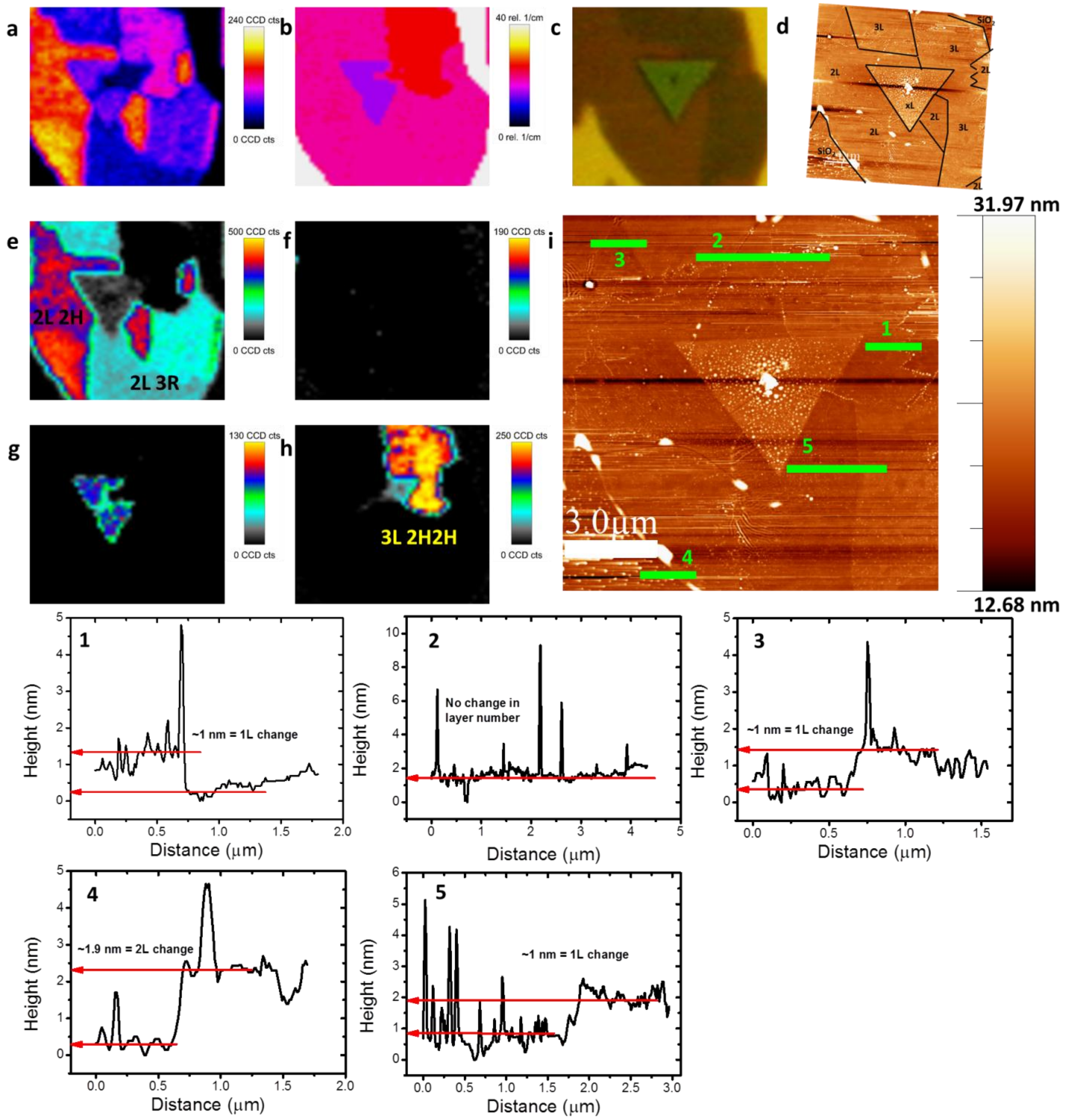

Figure S5 – (a) Peak intensity map over the range 10-50 cm^{-1} (b) Map of position of maximum peak intensity of the low-frequency Raman modes in the range of 0-40 cm^{-1} (c) Optical image of CVD $MoSe_2$ crystal from the area shown in Figures 4 and 5 in the main text (d) AFM scan with layer numbers overlaid as identified by Raman spectroscopy (e) Peak intensity map for 2L $MoSe_2$ SM at ~18 cm^{-1} (f) Peak intensity map for 2L $MoSe_2$ LBM at ~29 cm^{-1} (g) Peak intensity map for 3L $MoSe_2$ at ~13 cm^{-1} (h) Peak intensity map for 3L $MoSe_2$ at ~24 cm^{-1} (i) AFM height scan over $MoSe_2$ crystal with height profiles 1-5 labelled in overlay.

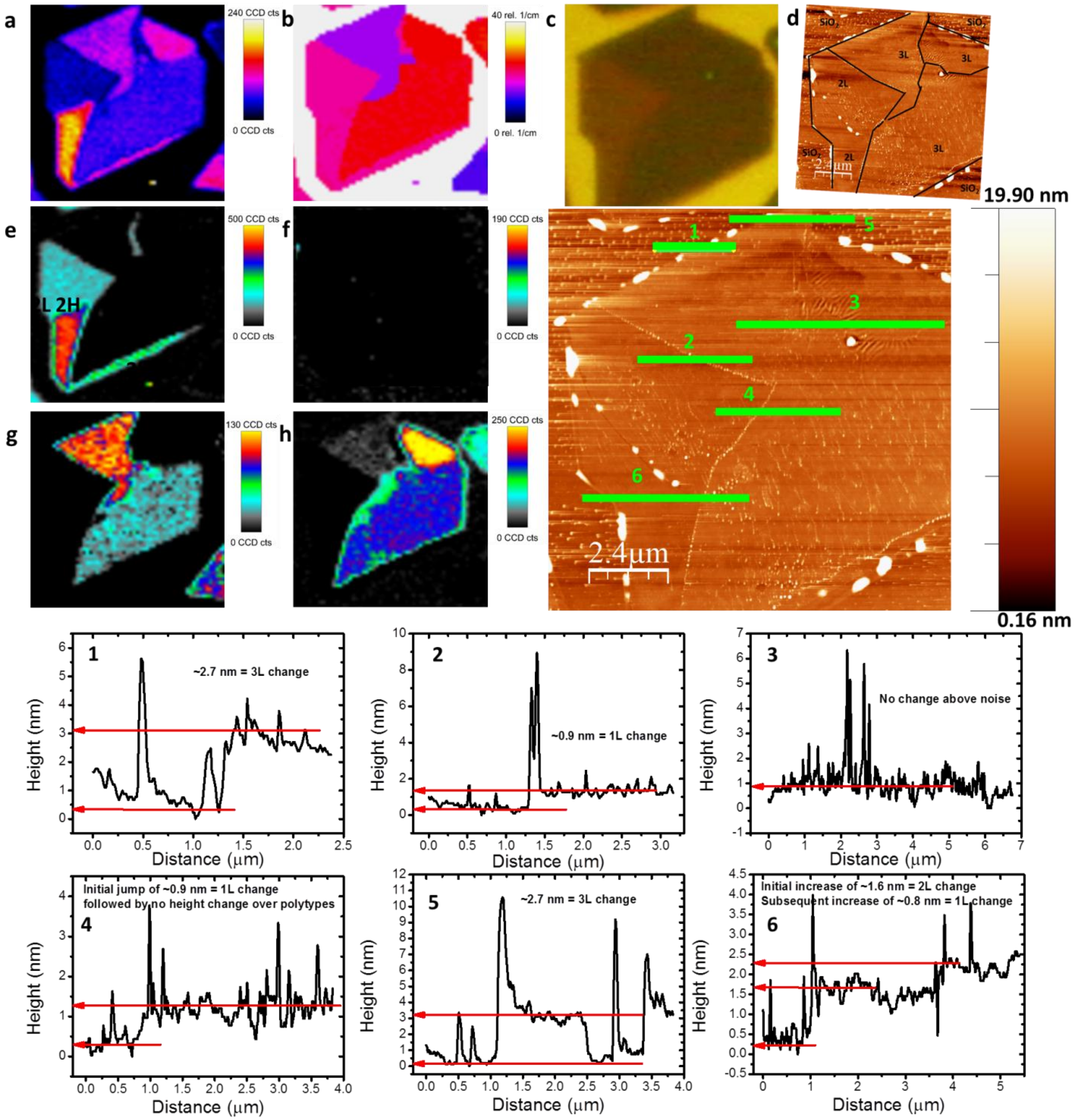

Figure S6 – (a) Peak intensity map over the range 10-50 cm^{-1} (b) Map of position of maximum peak intensity of the low-frequency Raman modes in the range of 0-40 cm^{-1} (c) Optical image of CVD $MoSe_2$ crystal from the area shown in Figures 4 and 5 in the main text (d) AFM scan with layer numbers overlaid as identified by Raman spectroscopy (e) Peak intensity map for 2L $MoSe_2$ SM at ~18 cm^{-1} (f) Peak intensity map for 2L $MoSe_2$ LBM at ~29 cm^{-1} (g) Peak intensity map for 3L $MoSe_2$ at ~13 cm^{-1} (h) Peak intensity map for 3L $MoSe_2$ at ~24 cm^{-1} (i) AFM height scan over $MoSe_2$ crystal with height profiles 1-6 labelled in overlay.

Additional WSe_2 Analysis

The peak position map for the A'_1/A_{1g} and $E'/E_g/E^1_{2g}$ overlapping mode is shown in Figure S7(a), with no discernible differences noted between different layer numbers. It is clear from correlation of Figure 6(b) in the main text with Figure S7(a) here that this peak maximum red-shifts to lower wavenumbers as layer number increases from 1 to 2+ layers, before blue-shifting to higher wavenumbers as layer number approaches bulk values. This is attributed to the enhancement of $E'/E_g/E^1_{2g}$ mode as layer number increases from 1 to 2+ layers, followed by the domination of the out-of-plane A'_1/A_{1g} mode as more layer numbers contribute in bulk-like regions, as is indicated in the spectra in Figure 6(d) of the main text. The *2LA(M)* mode peak position map is shown in Figure S7(b), with a notable blue shift between mono- and few-layer samples – however, this may simply be due to the enhanced relative intensity of the *2LA(M)* mode to the A'_1/A_{1g} and E'/E_g overlapping mode with increasing layer number. The PL position for monolayer appears at a wavelength of ~765 nm consistent with those previously reported for CVD-grown[11, 12] and mechanically exfoliated WSe_2[7, 13], showing an expected increase in wavelength with increasing layer number.

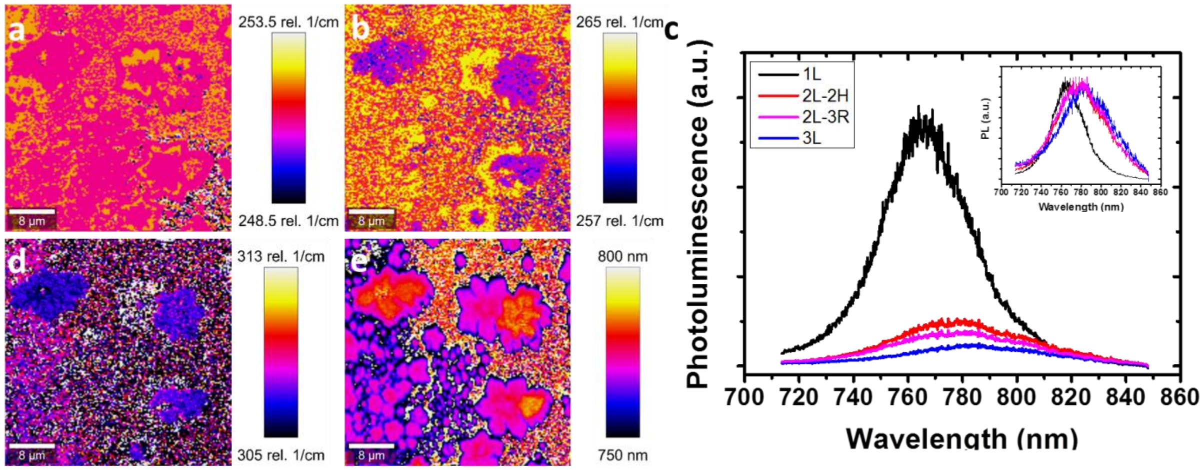

Figure S7 - Additional Raman and PL mapping of WSe_2 crystals of different layer thickness extracted from the regions shown in Figure 6(a) of the main text – (a) Position map of A'_1/A_{1g} and $E'/E_g/E^1_{2g}$ mode overlapping peak (b) Position map of *2LA(M)* mode (c) PL spectra of WSe_2 crystals of different layer thickness extracted from the regions shown in Figure 6(a) of the main text. Inset: Normalized version of the same spectra highlighting the evolution in peak position with changing layer thickness. (d) Position map of maximum B^1_{2g} mode (e) PL position map.

Additional WS_2 Analysis

A map of peak position for the combination mode centred at ~352 cm^{-1} is shown in Figure S8(a). This peak is a combination of contributions from the *2LA(M)* and $E'/E_g/E^1_{2g}$ modes that coincidentally overlap at this Raman shift. A red shift in position of maximum intensity with increasing layer number is evident, likely due to enhanced contributions with increasing layer number from the $E'/E_g/E^1_{2g}$ mode at a higher Raman shift than *2LA(M)*. A position maximum map of the A'_1/A_{1g} mode intensity maximum is shown in Figure S8(b). A blue shift in position of maximum intensity is clear as layer number increases. This is due to stronger interlayer contributions to the phonon restoring forces as layer number increases, resulting in a stiffening of the out-of-plane A'_1/A_{1g} mode, as the vibrations of this mode are more strongly affected by forces between the layers[14]. A peak intensity map of the zone-edge *LA(M)* phonon[14] is shown in Figure S8(c), with the corresponding spectra shown in Figure S8(h), which have been normalized to the 2LA(M) mode and offset for clarity. It is clear that this *LA(M)* mode has strongest intensity in

monolayer crystals, with a negligible change in position with increasing layer number. The behaviour of intensity with layer number in *LA(M)* can be compared to that of the low-frequency resonance peak discussed for WS_2 in the main text, and can be compared directly on the spectra here.

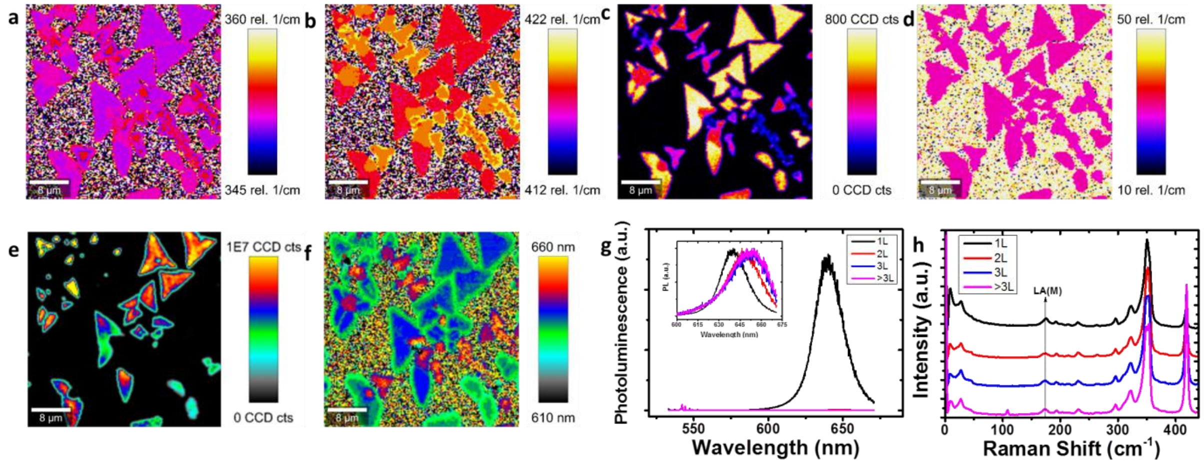

Figure S8 - Additional Raman and PL mapping of WS_2 crystals of different layer thickness extracted from the regions shown in the optical image in Figure 8(a) of the main text - (a) Position map of *2LA(M)* mode and $E'/E_g/E^1_{2g}$ mode overlap (b) Map of A'_1/A_{1g} mode position (c) Map of *LA(M)* mode intensity (d) Map of position of low-frequency mode at ~27 cm^{-1} (e) PL intensity map (f) Position map of PL maxima (g) Corresponding PL spectra of 1, 2, 3 and 3+L WS_2. Inset shows plots normalized to PL intensity (h) Spectra of 1, 2, 3 and 3+L WS_2 centred on the *LA(M)* mode.

Growth Schematic

The schematic in Figure S9(a) shows the quartz tube furnace configuration used for fabrication of CVD materials. A 10% H_2/Ar flow enters through the gas inlet as labelled. In Zone 2, sulfur or selenium solid precursors are placed, and evaporated at temperatures indicated. This vapour then flows downstream to microreactors placed in Zone 1, before being evacuated through the pump. A microreactor schematic is shown in Figure S9(b), as reported previously[2].

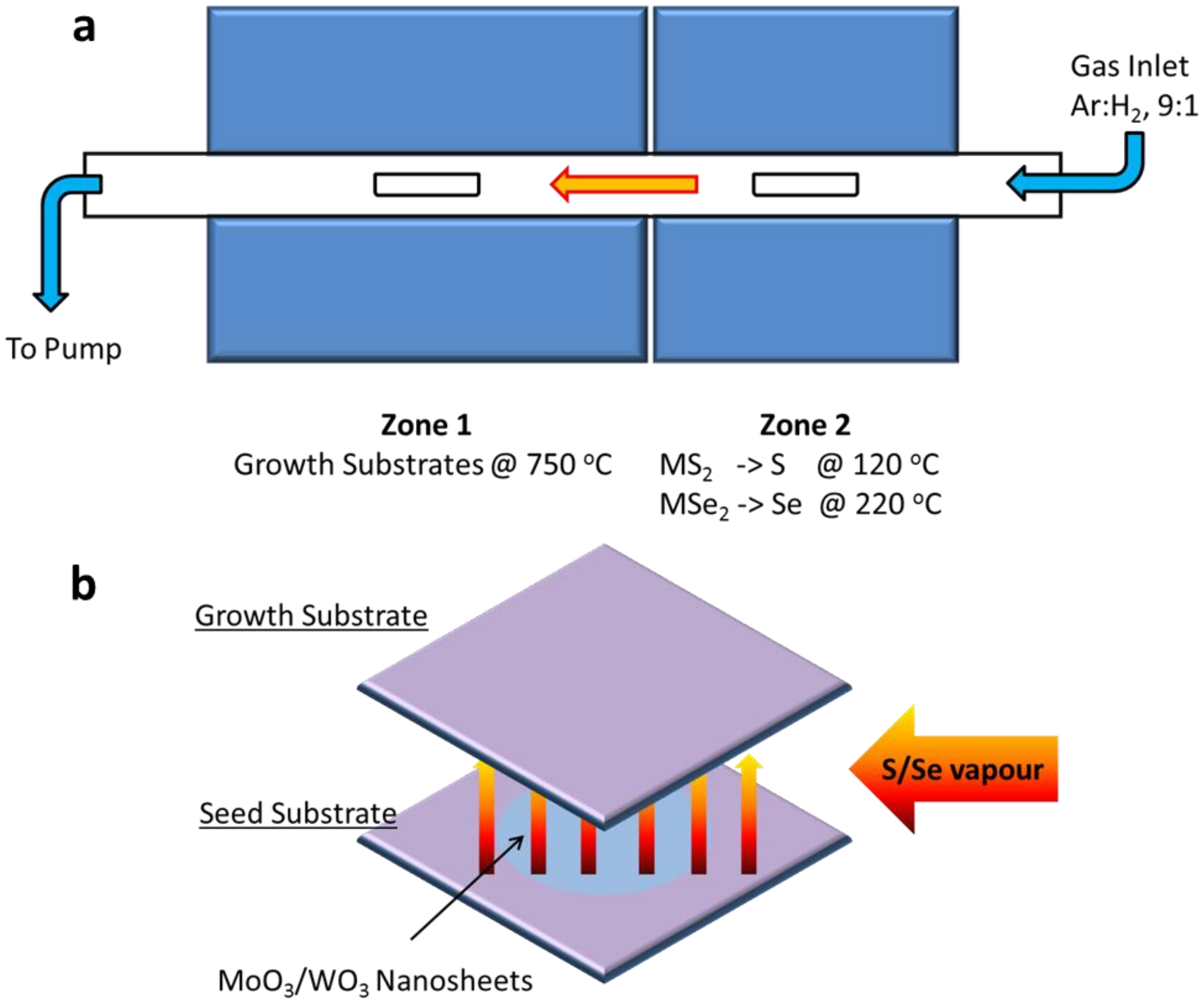

Figure S9 – (a) Schematic of furnace setup. Chalcogen powder is melted downstream and flowed through the microreactor (b) Schematic of CVD microreactor formed between the seed and target substrates, where sulfur reacts with MO_3 nanosheets to form MX_2 layers on the top substrate.

References

1. Lee, Y.H. et al. Synthesis of large-area MoS_2 atomic layers with chemical vapor deposition. *Adv Mater* **24**, 2320-2325 (2012).
2. O'Brien, M. et al. Transition Metal Dichalcogenide Growth via Close Proximity Precursor Supply. *Sci. Rep.* **4**, 7374 (2014).
3. van der Zande, A.M. et al. Grains and grain boundaries in highly crystalline monolayer molybdenum disulphide. *Nature materials* **12**, 554-561 (2013).
4. Liu, Z. et al. Strain and structure heterogeneity in MoS2 atomic layers grown by chemical vapour deposition. *Nat Commun* **5** (2014).
5. Gutierrez, H.R. et al. Extraordinary Room-Temperature Photoluminescence in Triangular WS_2 Monolayers. *Nano Lett* **13** 3447-3454 (2012).
6. Xia, J. et al. CVD synthesis of large-area, highly crystalline $MoSe_2$ atomic layers on diverse substrates and application to photodetectors. *Nanoscale* **6**, 8949-8955 (2014).
7. Tonndorf, P. et al. Photoluminescence emission and Raman response of monolayer MoS_2, $MoSe_2$, and WSe_2. *Opt. Express* **21**, 4908-4916 (2013).
8. Shaw, J. et al. Chemical vapor deposition growth of monolayer MoSe2 nanosheets. *Nano Res* **7**, 511-517 (2014).
9. Duan, X. et al. Lateral epitaxial growth of two-dimensional layered semiconductor heterojunctions. *Nat Nano* **9**, 1024-1030 (2014).
10. Gong, Y. et al. Vertical and in-plane heterostructures from WS_2/MoS_2 monolayers. *Nature materials* **13**, 1135-1142 (2014).
11. Huang, J.-K. et al. Large-Area Synthesis of Highly Crystalline WSe2 Monolayers and Device Applications. *ACS Nano* **8**, 923-930 (2014).
12. Kai, X. et al. Atomic-layer triangular WSe 2 sheets: synthesis and layer-dependent photoluminescence property. *Nanotechnology* **24**, 465705 (2013).
13. Zeng, H. et al. Optical signature of symmetry variations and spin-valley coupling in atomically thin tungsten dichalcogenides. *Sci. Rep.* **3** (2013).
14. Berkdemir, A. et al. Identification of individual and few layers of WS_2 using Raman Spectroscopy. *Scientific reports* **3**, 1755 (2013).
